# Editorial: Food cognition: the crossroads of psychology, neuroscience and nutrition

**DOI:** 10.3389/fnut.2023.1194053

**Published:** 2023-04-14

**Authors:** Carol Coricelli, Camille Rioux, Luisa Torri

**Affiliations:** ^1^German Institute of Human Nutrition Potsdam-Rehbrücke, Nuthetal, Germany; ^2^Deutsches Zentrum für Diabetes, Neuherberg, Germany; ^3^Charité-Universitätsmedizin Berlin, Corporate Member of Freie Universität Berlin, Humboldt-Universität zu Berlin and Berlin Institute of Health, Neuroscience Research Center, Berlin, Germany; ^4^Integrative Neuroscience and Cognition Center (INCC), Centre National de la Recherche Scientifique (CNRS), Université de Paris, Paris, France; ^5^University of Gastronomic Sciences, Pollenzo, Italy

**Keywords:** food cognition, eating behavior, cognitive neuroscience, decision-making, nutrition

Every day we make several food choices, some of which are better than others with regards to our physical and brain health (1). These decisions are based on various factors such as our primary needs to restore homeostasis in the body, reward mechanisms related to pleasure and higher-level goals, such as healthy or ethical diets (i.e., *vegetarianism, veganism, sustainable diets*). Food characteristics that influence our dietary choices have been categorized into (i) *basic* attributes, such as taste, and (ii) *abstract* attributes such as healthiness (2). The brain assigns value to these attributes while reaching the decision to ingest a certain food or not. Additionally, the nutritional composition of the foods that we ingest influences both our physical and brain health (3).

For a given food, such value can also vary depending on the individual's current metabolic and psycho-physiological states (i.e., *hunger*), memories, or environment in which the food occurs (4). Therefore, it is essential to approach the question of how food choices are made from a multidisciplinary perspective, including sensory science, nutrition, psychology, medicine, and neuroscience. The purpose of this Research Topic was to collate findings from these different disciplines to shed new light on the underlying brain mechanisms, how nutrition affects cognition and wellbeing across our lifespan in healthy and clinical populations.

Two reviews are presented as overviews to the topics covered. First, the opinion manuscript by Devoto et al., presents the external (environmental and food-specific) and internal (biological and psychological) factors guiding neural responses to food-cues. The presented model, expanded from a model originally proposed in the domain of drug addictions, may serve as a theoretical framework for experimental studies as well as the development of diagnostic tools and targeted clinical treatments for eating behaviors. Second, the review from Pearce et al. describes the mechanisms that underlie reinforcement learning and value-based decision making in the context of food choices. The authors argue that incorporating neurocognitive frameworks, such as *sign-* vs. *goal-tracking* phenotypes and *model-free* vs. *model-based* learning, can enhance our comprehension of eating behaviors like cravings, habits, and food addictions. Understanding the brain's responses to environmental food cues is essential, especially in Western societies, where obesogenic environments prevail.

Then the collection moves on to address how diet and nutrition impact our cognition. For example, Muth et al. present how a healthy diet and lifestyle act as a protective factor in contrasting negative mental health outcomes, reporting data from the crucial stressful times of COVID-19 pandemic. Food intake was tracked *via* the smartphone *FoodApp*, and the authors found that higher intakes of fruit and vegetable and physical activity during COVID-19 pandemic lockdowns were associated with higher mood levels and wellbeing in a German sample. In another study, Terenzi et al. show the role of social factors (i.e., *loneliness*) and dietary intake (measured *via* the *FoodApp*) during COVID-19 pandemic lockdowns in the development of conspiracy theories and psychotic-like experiences. Such subclinical symptoms were associated with lower fruit, carbohydrate, and iron intakes, as well as with higher fat intake.

The impact of eating behaviors on cognition has been studied in specific clinical populations, such as individuals with eating disorders, and during critical periods of life such as pregnancy or childhood, when individuals are learning about new foods.

For instance, an individual's weight status has a fundamental impact on their food intake, food preferences, and overall cognition and brain structures. Lakritz et al. have shown that individuals with Anorexia Nervosa and Orthorexia hold implicit associations between food variables that cue energy density (i.e., *processed foods*) and moral attributes differently than the general population. The moralization of food appears to be pervasive in such individuals, and such results present an important experimental and diagnostic tool less vulnerable to self-presentation and social desirability bias as explicit association measures. Foinant et al. show that children's food neophobia, namely the fear for new foods, influences how they represent different types of foods. Neophobic children, who tend to eat less fruit and vegetables, miscategorized foods as foods in a food/non-food categorization task compared to the neophilic counterparts. In the review manuscript by Waclawek and Park, it is shown that pregnancy represents a critical period in which changes in the endocrine, cognitive, and reward systems have been shown to take place. During pregnancy, alterations in metabolic modulations, dietary intake (maternal high-fat diets), and brain functioning (such as reduced gray matter, executive functions, and worse memory) represent an important model for understanding eating behaviors.

Additionally, the collection presents experimental findings that demonstrate the impact of environmental and social factors on food choices. For instance, Masento et al. found that vegetable intake of preschool children significantly changed across Italian, Polish and British samples, with the Polish sample having the highest number of portions of vegetables per day. The results suggest that healthy eating interventions for children must take into account the specific needs of the countries where they are implemented.

Moreover, DeJesus investigated parent judgments about foods for infants and found that parents rated foods they were familiar with as more appropriate for their infants. Additionally, the adults' own pickiness was related to what they would eat but not to what they would offer to infants, namely the adults would choose foods for the infants that they themselves would not consume. Such findings are important for social modeling behaviors such as adults demonstrating eating and actual liking of the offered foods to infants. Li et al. have shown that in China a higher nutritional literacy, which involves obtaining, understanding, and using accurate nutrition information to make healthy food choices, was linked to a lower prevalence of obesity among adolescents.

Finally, the collection concludes with a study emphasizing the significance of the evaluation context in which such food choices and behaviors are assessed. In Plaza et al. study, participants rated bread and pizza items of varying culinary preparation levels (e.g., homemade, ready-made, and a combination of the two) in a university cafeteria setting. The study employed both simple questions (*synthetic*) and questions with intensity attributes (*analytical*) to measure liking scores. The authors found that homemade pizza received lower liking scores (hedonic judgment) in the analytical task. The authors stress the fact that these findings highlight the importance of considering the evaluation task as part of the assessment context when designing ecologically valid consumer tests.

Taken together the work presented in our Research Topic shows that a multidisciplinary understanding of eating behaviors can lead to advancement in theoretical frameworks on food-related behaviors and can help in designing interventions promoting healthy and sustainable eating.

## Author contributions

CC and CR conceived the original idea of the Research Topic and wrote the Editorial. CC, CR, and LT reviewed and finalized the Editorial. All authors contributed to the article and approved the submitted version.
